# Supplementary figures and images for: Description of the microbiota in epidermal mucus and skin of sharks (Ginglymostoma cirratum and Negaprion brevirostris) and one stingray (Hypanus americanus)
Source: PeerJ. 2020 Dec 15;8:e10240. doi: 10.7717/peerj.10240 (PMC7747685; doi:10.7717/peerj.10240)

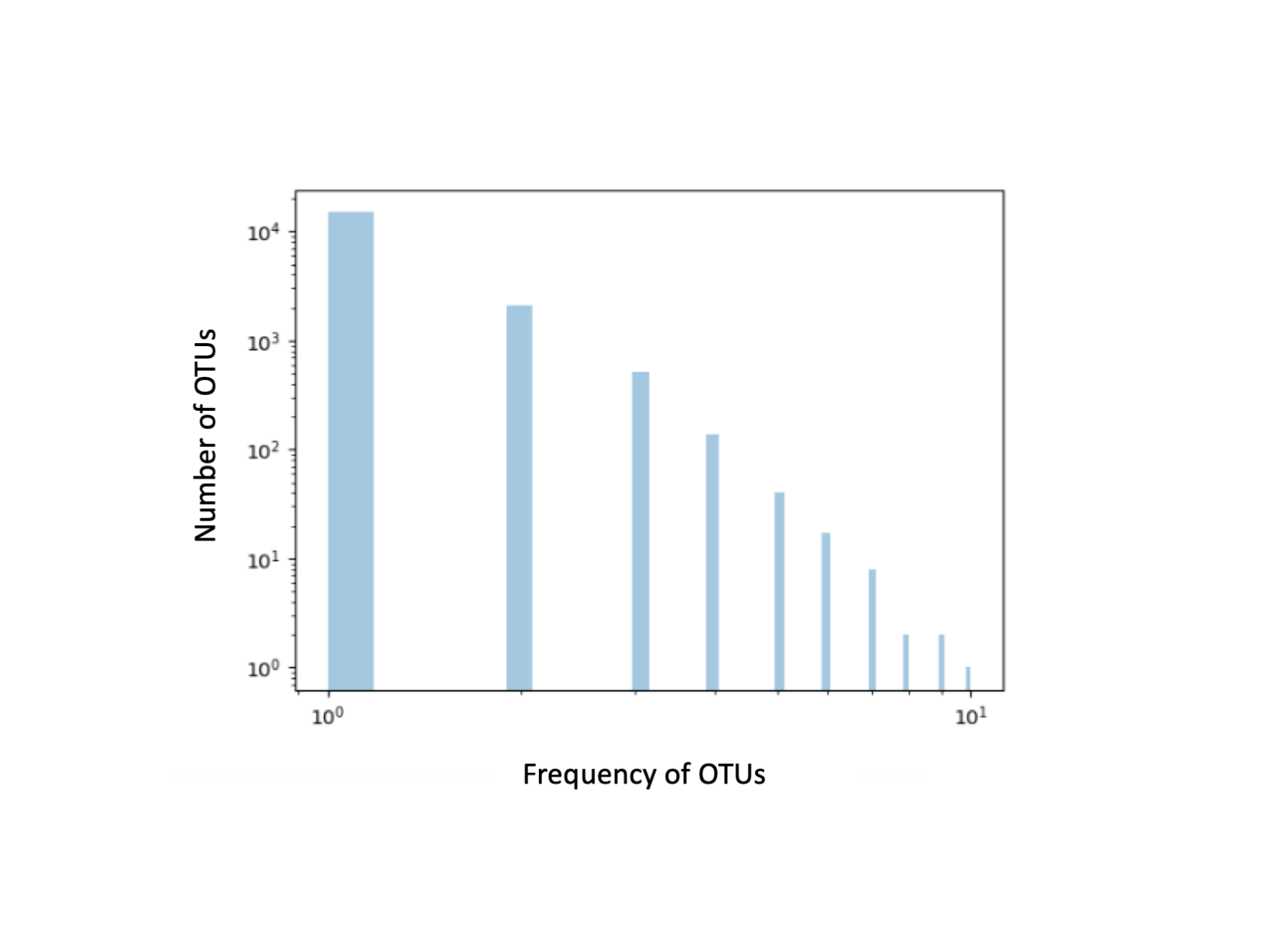

Supplement: Supplemental Information 1 [file peerj-08-10240-s001.png]

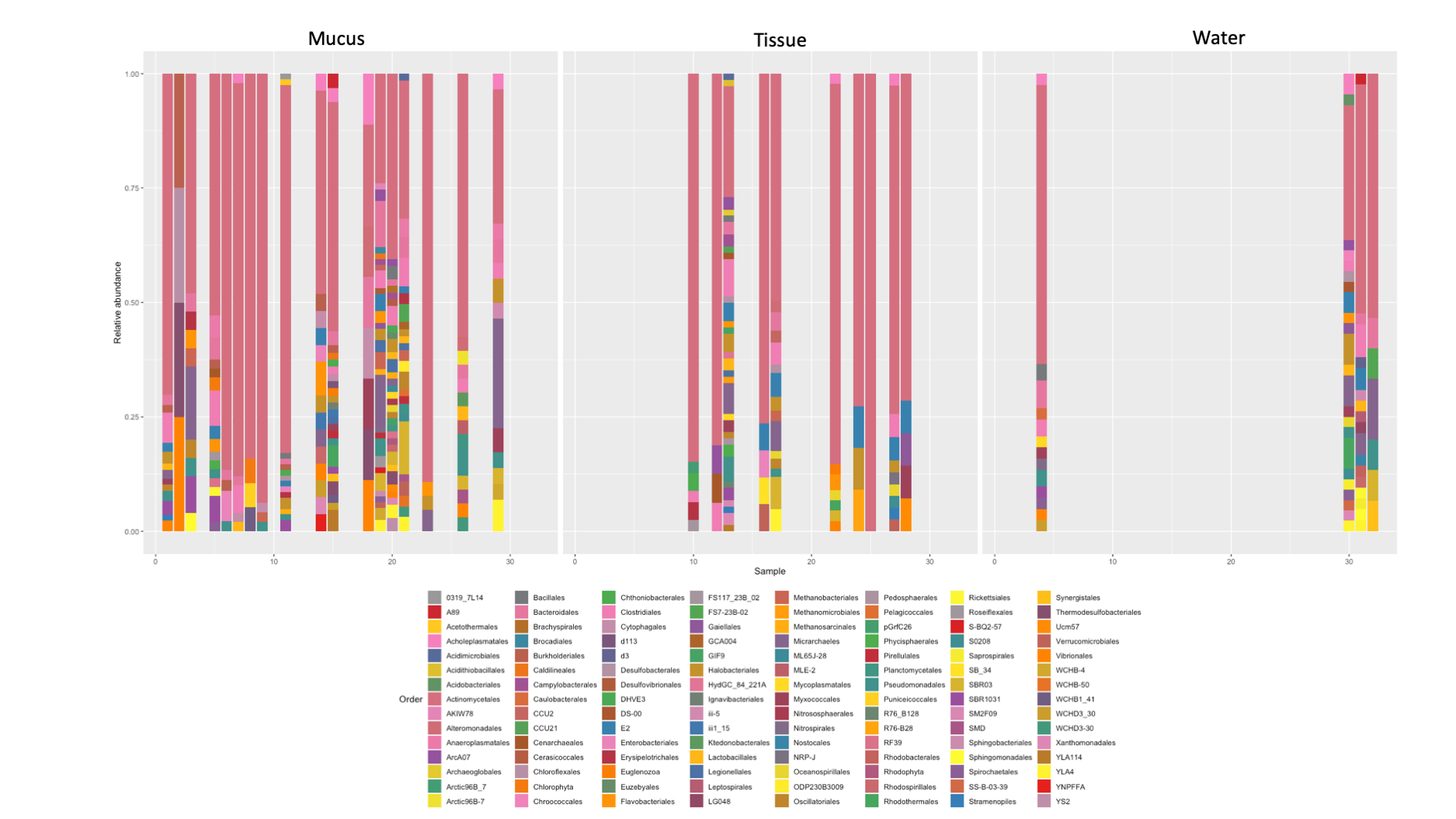

Supplement: Supplemental Information 2 [file peerj-08-10240-s002.png]

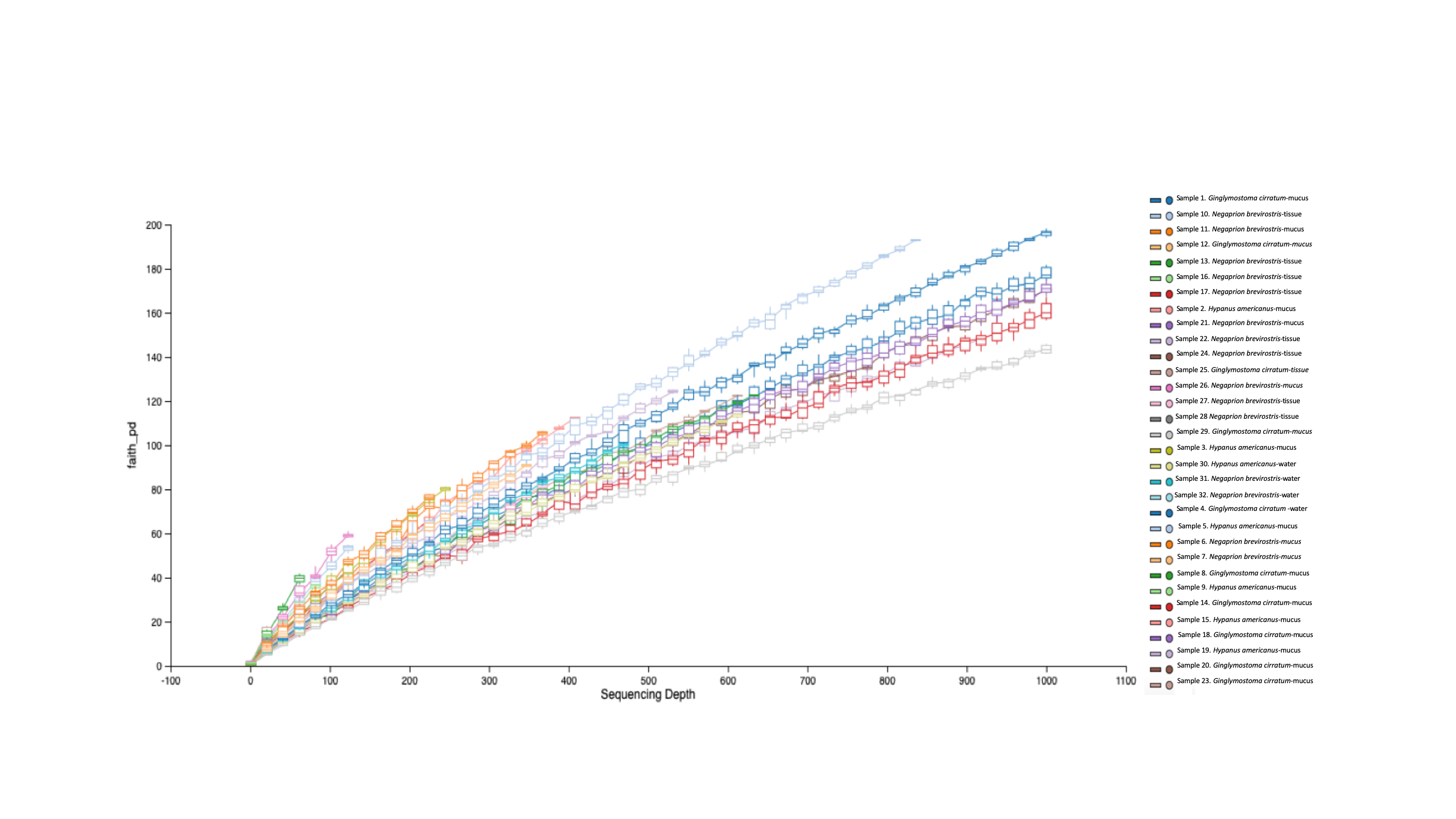

Supplement: Supplemental Information 3 — Growth rate of the Faiths pd in place of standard rarefaction curve, showing fast increase of diversity with sequencing depth per sample. [file peerj-08-10240-s003.png]

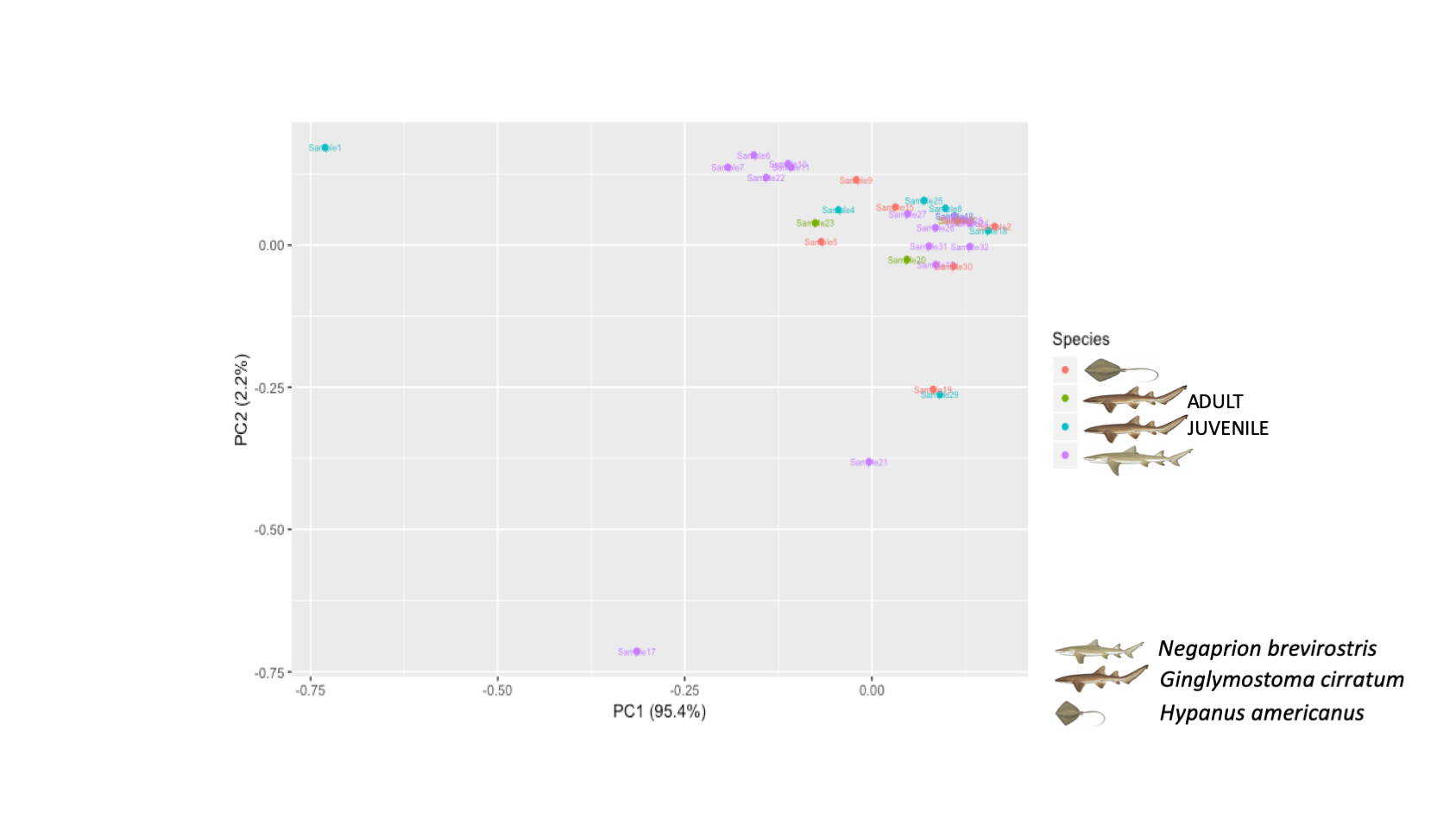

Supplement: Supplemental Information 4 — No clear pattern of similarity was observed among juvenile nurse shark od adult nurse shark samples. [file peerj-08-10240-s004.png]

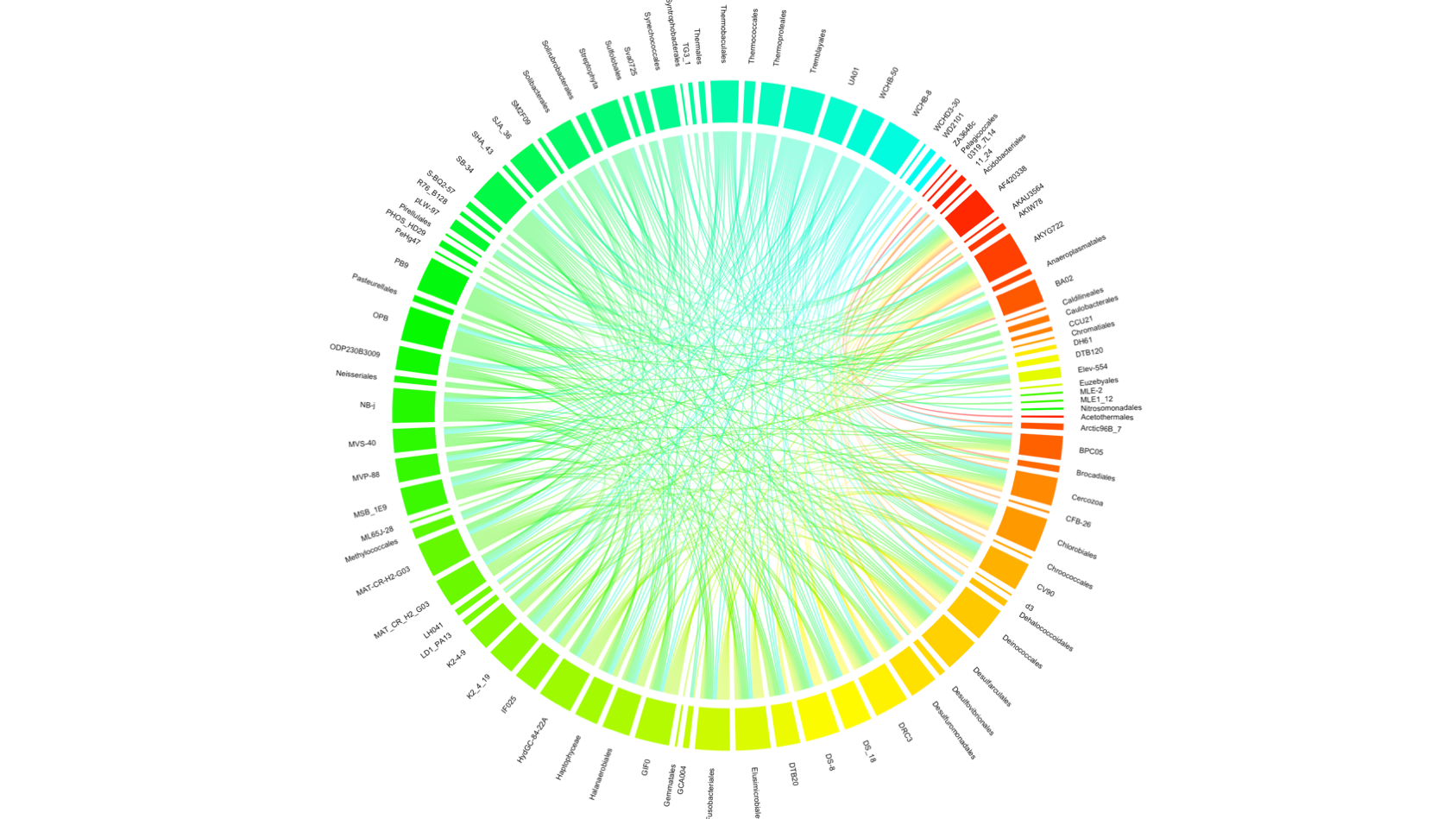

Supplement: Supplemental Information 5 [file peerj-08-10240-s005.png]
