# Supplementary material for: Description of the microbiota in epidermal mucus and skin of sharks (Ginglymostoma cirratum and Negaprion brevirostris) and one stingray (Hypanus americanus)
Source: PeerJ. 2020 Dec 15;8:e10240. doi: 10.7717/peerj.10240 (PMC7747685; doi:10.7717/peerj.10240)
Supplement: Supplemental Information 6 [file peerj-08-10240-s006.docx]

Supplementary Table 1: Information of samples used in library construction.

Sample ID Species Gender Age Sampling Site Type of Sample

Sample1 *Ginglymostoma cirratum* Male Juvenile Bimini, Bahamas Mucus

Sample2 *Hypanus americanus* Female Adult Bimini, Bahamas Mucus

Sample3 *Hypanus americanus*  - Adult Bimini, Bahamas Mucus

Sample4 *Ginglymostoma cirratum* Female Juvenile Bimini, Bahamas Water

Sample5 *Hypanus americanus* Female Adult Bimini, Bahamas Mucus

Sample6 *Negaprion brevirostris* - Adult Bimini, Bahamas Mucus

Sample7 *Negaprion brevirostris* - Juvenile Bimini, Bahamas Mucus

Sample8 *Ginglymostoma cirratum* Female Juvenile Bimini, Bahamas Mucus

Sample9 *Hypanus americanus* - Adult Bimini, Bahamas Mucus

Sample10 *Negaprion brevirostris* Female Juvenile Bimini, Bahamas Tissue

Sample11 *Negaprion brevirostris* - Adult Bimini, Bahamas Mucus

Sample12 *Ginglymostoma cirratum* Male Juvenile Bimini, Bahamas Tissue

Sample13 *Negaprion brevirostris* Female Juvenile Bimini, Bahamas Tissue

Sample14 *Ginglymostoma cirratum* Female Adult Islas Del Rosario Mucus

Sample15 *Hypanus americanus* - Adult Bimini, Bahamas Mucus

Sample16 *Negaprion brevirostris* Female Juvenile Bimini, Bahamas Tissue

Sample17 *Negaprion brevirostris* Female Juvenile Bimini, Bahamas Tissue

Sample18 *Ginglymostoma cirratum* Female Juvenile Bimini, Bahamas Mucus

Sample19 *Hypanus americanus* - Adult Bimini, Bahamas Mucus

Sample20 *Ginglymostoma cirratum* Female Adult Islas_Del_Rosario Mucus

Sample21 *Negaprion brevirostris* Female Juvenile Bimini, Bahamas Mucus

Sample22 *Negaprion brevirostris* Male Juvenile Bimini, Bahamas Tissue

Sample23 *Ginglymostoma cirratum* Female Adult Islas_Del_Rosario Mucus

Sample24 *Negaprion brevirostris* Male Juvenile Bimini, Bahamas Tissue

Sample25 *Ginglymostoma cirratum* Male Juvenile Bimini, Bahamas Tissue

Sample26 *Negaprion brevirostris* Female Juvenile Bimini, Bahamas Mucus

Sample27 *Negaprion brevirostris* Female Juvenile Bimini, Bahamas Tissue

Sample28 *Negaprion brevirostris* Female Juvenile Bimini, Bahamas Tissue

Sample29 *Ginglymostoma cirratum* Female Juvenile Bimini, Bahamas Mucus

Sample30 *Hypanus americanus* - Adult Bimini, Bahamas Water

Sample31 *Negaprion brevirostris* - Juvenile Bimini, Bahamas Water

Sample32 *Negaprion brevirostris* Female Juvenile Bimini, Bahamas Water
