# Supplementary material for: Description of the microbiota in epidermal mucus and skin of sharks (Ginglymostoma cirratum and Negaprion brevirostris) and one stingray (Hypanus americanus)
Source: PeerJ. 2020 Dec 15;8:e10240. doi: 10.7717/peerj.10240 (PMC7747685; doi:10.7717/peerj.10240)
Supplement: Supplemental Information 7 [file peerj-08-10240-s007.docx]

Supplementary Table 2. Percentage of orders found in the total number of reads analyzed in this study.

| Order | % Order found in the total number of analyzed reads |
| --- | --- |
| Brachyspirales | 0,041 |
| Cerasicoccales | 0,122 |
| Chthoniobacterales | 0,041 |
| Leptospirales | 0,081 |
| Micrarchaeles | 1,985 |
| Pedosphaerales | 0,122 |
| Pelagicoccales | 0,162 |
| Rhodothermales | 0,284 |
| Roseiflexales | 0,162 |
| Saprospirales | 0,122 |
| 0319_7L14 | 0,041 |
| 04A5 | 0,122 |
| 11_24 | 0,041 |
| A89 | 0,122 |
| Acetothermales | 0,041 |
| Acholeplasmatales | 0,729 |
| Acidimicrobiales | 0,162 |
| Acidithiobacillales | 0,081 |
| Acidobacteriales | 0,122 |
| Actinomycetales | 50,932 |
| AF420338 | 0,041 |
| AKAU3564 | 0,081 |
| AKIW78 | 0,041 |
| AKYG722 | 0,041 |
| Alteromonadales | 0,527 |
| Anaeroplasmatales | 0,567 |
| ArcA07 | 0,608 |
| Archaeoglobales | 0,122 |
| Arctic96B_7 | 0,081 |
| Arctic96B-7 | 0,041 |
| BA02 | 0,041 |
| Bacillales | 0,608 |
| Bacteroidales | 2,35 |
| BPC05 | 0,041 |
| Brachyspirales | 0,041 |
| Brocadiales | 0,041 |
| Burkholderiales | 0,729 |
| Caldilineales | 0,243 |
| Campylobacterales | 0,365 |
| Caulobacterales | 0,081 |
| CCU2 | 0,365 |
| CCU21 | 0,041 |
| Cenarchaeales | 0,203 |
| Cerasicoccales | 0,041 |
| Cercozoa | 0,041 |
| CFB-26 | 0,081 |
| Chlamydiales | 0,081 |
| Chlorobiales | 0,041 |
| Chloroflexales | 0,081 |
| Chlorophyta | 0,243 |
| Chromatiales | 0,122 |
| Chroococcales | 0,122 |
| Chthoniobacterales | 0,243 |
| Clostridiales | 3,404 |
| CV90 | 0,041 |
| Cytophagales | 0,365 |
| d113 | 0,122 |
| d3 | 0,162 |
| Dehalococcoidales | 0,041 |
| Deinococcales | 0,041 |
| Desulfarculales | 0,081 |
| Desulfobacterales | 0,284 |
| Desulfovibrionales | 0,041 |
| Desulfuromonadales | 0,041 |
| DH61 | 0,041 |
| DHVE3 | 0,081 |
| DRC3 | 0,041 |
| DS_18 | 0,041 |
| DS-00 | 0,081 |
| DS-8 | 0,041 |
| DTB120 | 0,041 |
| DTB20 | 0,041 |
| E2 | 1,702 |
| Elev-554 | 0,041 |
| Elusimicrobiales | 0,041 |
| Enterobacteriales | 0,689 |
| Erysipelotrichales | 0,527 |
| Euglenozoa | 0,203 |
| Euzebyales | 0,041 |
| Flavobacteriales | 0,972 |
| FS117_23B_02 | 0,284 |
| FS7-23B-02 | 0,284 |
| Fusobacteriales | 0,041 |
| Gaiellales | 0,122 |
| GCA004 | 0,243 |
| Gemmatales | 0,162 |
| GIF0 | 0,041 |
| GIF9 | 0,081 |
| H39 | 0,203 |
| Halanaerobiales | 0,041 |
| Halobacteriales | 1,661 |
| Haptophyceae | 0,041 |
| HydGC_84_221ª | 0,081 |
| HydGC-84-22ª | 0,041 |
| IF025 | 0,041 |
| Ignavibacteriales | 0,041 |
| iii-5 | 0,203 |
| iii1_15 | 0,162 |
| K2_4_19 | 0,041 |
| K2-4-9 | 0,041 |
| Ktedonobacterales | 0,081 |
| Lactobacillales | 0,81 |
| LD1_PA13 | 0,041 |
| Legionellales | 0,446 |
| Leptospirales | 0,041 |
| LG048 | 0,122 |
| LH041 | 0,041 |
| MAT_CR_H2_G03 | 0,041 |
| MAT-CR-H2-G03 | 0,041 |
| Methanobacteriales | 0,729 |
| Methanocellales | 0,081 |
| Methanomicrobiales | 0,162 |
| Methanosarcinales | 0,284 |
| Methylococcales | 0,041 |
| Micrarchaeles | 0,77 |
| ML65J-28 | 0,041 |
| MLE-2 | 0,122 |
| MLE1_12 | 0,081 |
| MSB_1E9 | 0,041 |
| MSBL9 | 0,081 |
| MVP-88 | 0,041 |
| MVS-40 | 0,041 |
| Mycoplasmatales | 0,243 |
| Myxococcales | 0,77 |
| NB-j | 0,041 |
| Neisseriales | 0,041 |
| Nitrosomonadales | 0,081 |
| Nitrososphaerales | 0,122 |
| Nitrospirales | 0,365 |
| Nostocales | 0,081 |
| NRP-J | 0,081 |
| Oceanospirillales | 0,567 |
| ODP230B3009 | 0,122 |
| OPB | 0,041 |
| Oscillatoriales | 0,77 |
| Pasteurellales | 0,041 |
| PB9 | 0,041 |
| Pedosphaerales | 0,081 |
| PeHg47 | 0,081 |
| pGrfC26 | 0,162 |
| PHOS_HD29 | 0,041 |
| Phycisphaerales | 0,284 |
| Pirellulales | 0,243 |
| Planctomycetales | 0,122 |
| pLW-97 | 0,041 |
| Pseudanabaenales | 0,041 |
| Pseudomonadales | 1,54 |
| Puniceicoccales | 0,162 |
| R76_B128 | 0,041 |
| R76-B28 | 0,041 |
| RB41 | 0,081 |
| RF39 | 0,324 |
| Rhizobiales | 0,162 |
| Rhodobacterales | 0,203 |
| Rhodophyta | 0,081 |
| Rhodospirillales | 0,203 |
| Rhodothermales | 0,122 |
| Rickettsiales | 0,365 |
| Roseiflexales | 0,041 |
| S-BQ2-57 | 0,041 |
| S0208 | 0,162 |
| Saprospirales | 0,081 |
| SB_34 | 0,041 |
| SB-34 | 0,041 |
| SBR03 | 1,742 |
| SBR1031 | 0,972 |
| SBYZ_6080 | 0,081 |
| SHA_43 | 0,041 |
| SJA_36 | 0,041 |
| SM2F09 | 0,041 |
| SMD | 0,162 |
| Solibacterales | 0,041 |
| Solirubrobacterales | 0,041 |
| Sphingobacteriales | 0,081 |
| Sphingomonadales | 0,284 |
| Spirochaetales | 0,324 |
| SS-B-03-39 | 0,122 |
| Stramenopiles | 0,324 |
| Streptophyta | 0,081 |
| Sulfolobales | 0,162 |
| Sva0725 | 0,041 |
| Synechococcales | 0,041 |
| Synergistales | 0,203 |
| Syntrophobacterales | 0,122 |
| TG3_1 | 0,041 |
| Thermales | 0,162 |
| Thermobaculales | 0,041 |
| Thermococcales | 0,041 |
| Thermodesulfobacteriales | 0,446 |
| Thermoplasmatales | 0,041 |
| Thermoproteales | 0,041 |
| Thiotrichales | 0,122 |
| Tremblayales | 0,041 |
| UA01 | 0,041 |
| Ucm1571 | 0,162 |
| Ucm57 | 0,162 |
| Verrucomicrobiales | 0,567 |
| Vibrionales | 0,891 |
| WCHB-4 | 0,243 |
| WCHB-50 | 0,122 |
| WCHB-8 | 0,041 |
| WCHB1_41 | 0,081 |
| WCHD3_30 | 0,243 |
| WCHD3-30 | 0,284 |
| WD2101 | 0,041 |
| Xanthomonadales | 0,284 |
| YLA114 | 0,243 |
| YLA4 | 1,053 |
| YNPFFA | 0,041 |
| YS2 | 0,122 |
| ZA3648c | 0,041 |
