# Supplementary material for: Description of the microbiota in epidermal mucus and skin of sharks (Ginglymostoma cirratum and Negaprion brevirostris) and one stingray (Hypanus americanus)
Source: PeerJ. 2020 Dec 15;8:e10240. doi: 10.7717/peerj.10240 (PMC7747685; doi:10.7717/peerj.10240)
Supplement: Supplemental Information 8 — (a) Normality tests to evaluate diversity indices. (A) refers to adults and (J) to juveniles. (b) Test to evaluate potential significant differences between diversity indices for (1) species and (2) type of sample. [file peerj-08-10240-s008.docx]

Supplementary Table 3. (a) Normality tests to evaluate diversity indices. (A) refers to adults and (J) to juveniles. (b) Test to evaluate potential significant differences between diversity indices for (1) species and (2) type of sample.

(a)

| Shapiro-Wilk | | |
| --- | --- | --- |
| species | Simpson | Shannon |
| *N_brevirostris* | 0.02243 | 6.46E-05 |
| *G_cirratum (A)* | 0.2252 | 0.7718 |
| *G_cirratum(J)* | 0.4487 | 0.1559 |
| *H_americanus* | 0.003559 | 0.3835 |

(b)

(1)

| Kruskal-Wallis by species (p-value) | |
| --- | --- |
| Simpson | 0.3311 |
| Shannon | 0.3312 |

(2)

| Kruskal-Wallis by type of sample (p-value) | |
| --- | --- |
| Simpson | 0.3253 |
| Shannon | 0.3254 |
