# Supplementary material for: Description of the microbiota in epidermal mucus and skin of sharks (Ginglymostoma cirratum and Negaprion brevirostris) and one stingray (Hypanus americanus)
Source: PeerJ. 2020 Dec 15;8:e10240. doi: 10.7717/peerj.10240 (PMC7747685; doi:10.7717/peerj.10240)
Supplement: Supplemental Information 9 — Orange corresponds to bacteria considered as fish pathogens; pink, for other animal pathogens; blue for fish symbionts; purple, for other animal symbionts; and green, for environmental bacteria. Characteristics for each genus and species determined comes from the Bergey’s Manual of Systematics of Archaea or from sources stated. [file peerj-08-10240-s009.doc]

Supplementary Table 4: Bacteria found in samples of mucus, skin and water from sharks and rays. Orange corresponds to bacteria considered as fish pathogens; pink, for other animal pathogens; blue for fish symbionts; purple, for other animal symbionts; and green, for environmental bacteria. Characteristics for each genus and species determined comes from the Bergey´s Manual of Systematics of Archaea or from sources stated.

| Phylum | Class | Order | Genus and species (if determined) | Type of sample (this study) | Characteristics |
| --- | --- | --- | --- | --- | --- |
|  |  |  |  |  |  |
| Crenarchaeota | Thermoprotei | Thermoproteales |  | Mucus and skin | Extremophile environmental bacteria |
| Actinobacteria |  | Actinomycetales | *Arcanobacterium* | Mucus and skin | Environmental and aquatic bacteria. Some species of this group have been isolated from marine mammals [1] |
|  |  |  | *Cellulisimicrobium* |  | *Mycobacterium, Nocardia* and *Rhodococcus* have been reported as fish pathogens [2–4] |
|  |  |  | *Corvenebacterium* |  |  |
|  |  |  | *Glycomyces* |  |  |
|  |  |  | *Gordonia* |  |  |
|  |  |  | *Haloactinospora* |  |  |
|  |  |  | *Microbacterium* |  |  |
|  |  |  | *Mycetocola* |  |  |
|  |  |  | *Mycrococcus* |  |  |
|  |  |  | *Rothia* |  |  |
|  |  |  | *Mycobacterium* |  |  |
|  |  |  | *Nocardia* |  |  |
|  |  |  | *Rhodococcus* |  |  |
|  |  |  | *Nocardioides* |  |  |
|  |  |  | *Pseudonorcardia* |  |  |
|  |  |  | *Streptomonospora* |  |  |
|  |  |  | *Streptomyces* |  |  |
|  |  |  | *Rhodococcus ruber* | Water | *Rhodococcus ruber* is an environmental bacteria |
|  |  |  | *Propionibacterium acnes* |  | *Propionibacterium acnes has been reported as normal human skin microbiota* |
|  |  |  |  |  |  |
|  |  | Euzebyales | *Euzebya* | Mucus and skin | Previously isolated from a sea cucumber [5] |
|  |  | Gaiellales |  | Mucus and skin | Described from deep mineral water [6] |
|  |  | Solirubrobacterales |  | Mucus and skin | Environmental bacteria |
| Acidobacteria | Chloracidobacteria |  |  | Water | Environmental bacteria |
|  | Koribacteraceae |  |  | Mucus and skin | Environmental bacteria |
|  | Solibacterales |  |  | Mucus and skin | Environmental bacteria |
| Bacteroidetes |  | Bacteroidales | *Bacteroides*  *Prevotella copri* | Mucus and skin | Some species of *Bacteroides* have been described as human pathogens in periodontal disease and *Prevotella copri* has been found as pathogen in intestinal inflammation. |
|  |  |  | *Butyricimonas* |  | *Butyricimonas* has been found as normal beneficial intestinal microbiota in humans [7] |
|  |  |  | *Parabacteroides gordonii* |  | *Parabacteroides gordonii* has been isolated from human blood cultures [8] |
|  |  |  |  |  |  |
|  |  | Bacteroidales | *Bacteroides caccae* | Water | Human intestinal microbiota |
|  |  |  | *Bacteroides fragilis* |  |  |
|  |  |  | *Bacteroides uniformis* |  |  |
|  |  | Cytophagales | *Hymenobacter* | Mucus and skin | Described as environmental bacteria, in soil, water and ice |
|  |  |  | *Flammeovirga* |  |  |
|  |  | Flavobacteriales | *Sulcia muelleri* | Mucus and skin | *Sulcia muelleri* has been described in symbiotic association with insects [9] |
|  |  |  | *Flavobacterium* |  | *Flavobacterium* has been described as a commensal in fish, showing antimicrobial activity against fish pathogens such as *Vibrio* sp. [10] |
|  |  |  |  |  |  |
|  |  |  |  |  |  |
|  |  | Flavobacteriales | *Fluviicola* | Water | *Fluviicola, Aequorivita, Aquimarina, Chryseobacterium* and *Bizionia* have been described as environmental bacteria, found in seawater and soil |
|  |  |  | *Aequorivita* |  |  |
|  |  |  | *Aquimarina* |  |  |
|  |  |  | *Bizionia* |  |  |
|  |  |  | *Chryseobacterium* |  |  |
|  |  |  |  |  |  |
|  |  | Rhodotermales | *Salisaeta* | Mucus and skin | Halophilic bacteria |
|  |  | Saprospirales | *Chitinophaga* | Water | Environmental bacteria |
|  |  | Sphingobacteriales |  | Mucus and skin | Environmental bacteria |
| Chlamydiae |  |  |  | Mucus and skin | Pathogens for birds and mammals |
| Chlorobi |  | Chlorobiales | *Chlorobium chlorochromatii* | Water | Photosynthetic aquatic bacteria |
|  |  | Ignavibacteriales |  | Water | Environmental bacteria isolated from hot Springs |
| Chloroflexi |  | Caldilineales |  | Water | Environmental bacteria |
|  |  | Chloroflexales |  | Water | Photosynthetic aquatic bacteria |
|  |  | Roseiflexales | *Kouleothrix* | Water | Environmental bacteria |
|  |  |  | *Roseiflexus* |  |  |
|  |  | Ktedonobacterales |  | Water | Environmental bacteria |
|  |  | Thermobaculales |  | Water | Environmental bacteria |
| Cyanobacteria |  | Nostocales |  | Water |  |
|  |  | Chroocoocales |  | Water |  |
|  |  | Oscillatoriales | *Microcoleus* | Mucus and skin |  |
|  |  | Pseudanabaenales | *Leptolyngbya* | Mucus and skin |  |
|  |  | Synecchococcales | *Synechococcus* | Mucus and skin | Environmental marine bacteria |
| Elusimicrobia |  | Elusimicrobiales |  | Mucus and skin | Marine bacteria |
| Fibrobacteres |  |  |  | Mucus and skin | Celullose degrading bacteria |
| Firmicutes |  | Bacillales | *Bacillus megaterium* | Mucus and skin | Environmental bacteria. *Bacillus polymyxa* has been isolated from fish guts. Some strains produce antibiotics [11]. *Bacillus subtilis* has been suggested as a probiotic to improve fish feeding [12]. |
|  |  |  | *Bacillus polymyxa* |  | *Staphylococcus* bacteria are commonly found in mucus and skin of mammals and birds. |
|  |  |  | *Bacillus subtilis* |  |  |
|  |  |  | *Bacillus licheniformes* |  |  |
|  |  |  | *Marinococcus* |  |  |
|  |  |  | *Cohnella* |  |  |
|  |  |  | *Staphylococcus* |  |  |
|  |  | Lactobacillales | *Alloiococcus* | Mucus and skin | *Enterococcus cecorum* has been reported as a pathogen in chickens [13]. |
|  |  |  | *Desemzia* |  | *Granulicatella* has been reported as normal microbiota of the human oral cavity*.* |
|  |  |  | *Granulicatella* |  | *Alloiococcus* is found in human middle ear fluid [14]. |
|  |  |  | *Enterococcus cecorum* |  | *Lactobacillus iners* |
|  |  |  | *Lactobacillus iners* |  | *and Lactobacillus zeae* have been found to be protective for nemotades [15] and mammals |
|  |  |  | *Lactobacillus zeae* |  |  |
|  |  |  |  |  |  |
|  |  | Lactobacillales | *Streptococcus parauberis* | Water | *Streptococcus parauberis* has been identified as a fish pathogen [16] |
|  |  | Clostridiales | *Caldicoprobacter* | Mucus and skin | *Caldicoprobacter* |
|  |  |  | *Gracilibacter* |  | *Gracilibacter* |
|  |  |  | *Garciella* |  | *Garciella, Pelotomaculum, Sulfobacillus,* and *Tepidimicrobium* were obtained from hot springs. |
|  |  |  | *Butyrivibrio* |  | *Desulfosporosinus meridiei* and *Pelosinus* have been previously isolated from groundwater |
|  |  |  | *Desulfosporosinus meridiei* |  | *Butyrivibrio* and *Selenomonas* are found in the gastrointestinal tract of ruminants*.* |
|  |  |  | *Pelotomaculum Faecalibacterium prausnitzii* |  |  |
|  |  |  | *Ruminococcus Sulfobacillus Helcococcus Peptoniphilus Tepidimicrobium Megamonas* |  | *Faecalibacterium prausnitzii, Peptoniphilus, Ruminococcus, Megamonas* are important bacteria in the human gut microbiota |
|  |  |  | *Pelosinus* |  | *Helcoccocus* is a pathogen in humans [17]. |
|  |  |  | *Selenomonas* |  | *Sporomusa* has been actively investigated for biofuel production [18]. |
|  |  |  | *Sporomusa* |  | *Epulopiscium* has been described as fish endosymbiont [19]. |
|  |  |  | *Epulopiscium* |  |  |
|  |  | Clostridiales | *Clostridiisalibacter* | Water | Environmental halophilic bacteria |
|  |  |  |  |  |  |
|  |  | Halanaerobiales |  | Mucus and skin | Environmental halophilic bacteria |
|  |  | Erysipelotrichales | *Catenibacterium mitsuokai* | Mucus and skin | *Catenibacterium mitsuokai* and *Coprobacillus* has been isolated from the human gut |
|  |  |  | *Coprobacillus* |  |  |
|  |  |  | *Erysipelothrix* |  | *Erysipelothrix* is considered an environmental bacteria, sometimes pathogenic |
|  |  |  | *Sharpea* |  | *Sharpea* was isolated from horse faeces [20]. |
|  |  |  |  |  |  |
| Fusobacteria |  | Fusobacteriales | *Fusobacterium* | Mucus and skin | Present in the human gut [46]. |
| Gemmatimonadetes |  |  |  | Mucus and skin | Environmental bacteria |
| Nitrospirae |  | Nitrospirales | *Leptospirillum ferrodiazotrophum* | Mucus and skin | *Leptospirillum ferrodiazotrophum* is a Nitrogen fixing bacterium found in soil. *Thermodesulfovibrio* is a termophilic baterium found in wastewater. |
|  |  |  | *Thermodesulfovibrio* |  |  |
| Nitrospirae |  | Nitrospirales | *Nitrospira* | Water | Nitrite-oxidizing bacteria found in marine water |
| Planctomycetes |  | Brocadiales |  | Mucus and skin | Marine environmental bacteria. Described as anaerobic ammonium oxidative bacteria (anammox). |
|  |  | Gemmatales |  | Mucus and skin | Environmental bacteria |
|  |  | Pirellulales | *Planctomyces* | Mucus and skin | Aquatic bacteria |
|  |  | Phycisphaerales |  | Water | Previously isolated from marine algae [21]. |
| Proteobacteria |  | Rhizobiales | *Agrobacterium sullae* | Water | Soil bacteria |
|  |  | Rhodobacterales | *Rhodovulum Rubellimicrobium* | Mucus and skin | Environmental bacteria |
|  |  |  |  |  |  |
|  |  | Rhodospirillales | *Skermanella* | Mucus and skin | Environmental bacteria |
|  |  | Sphingomonadales | *Sphingomonas* | Water | Environmental bacteria |
|  |  | Burkholderiales | *Sutterella* | Mucus and skin | *Sutterella* has been isolated from the human gut. |
|  |  |  | *Burkholderia Comamonas* |  | *Janthinobacterium lividum* has been found in the skin of some amphibians and appears to prevent infection by *Batrachochytrium dendrobatidis* [22] |
|  |  |  | *Thiomonas* |  | *Burkholderia, Comamonas Thiomonas, Cupriavidus,* and *Herminiimonas* are |
|  |  |  | *Cupriavidus* |  | environmental bacteria from water, soil and ice |
|  |  |  | *Herminiimonas Janthinobacterium lividum* |  |  |
|  |  |  |  |  |  |
|  |  | Nitrosomodales |  | Mucus and skin | Marine bacteria |
|  |  | Neisseriales | *Neisseria cinerea* | Water | Non-pathogenic bacteria associated with normal microbiota of different animals |
|  |  | Trembayales | *Carsonella ruddii* | Mucus and skin | Described as endosymbionts of insects [23]. |
|  |  |  |  |  |  |
|  |  |  |  |  |  |
|  |  | Desulfovibrionales | *Desulfonatronovibrio* | Mucus and skin | Aquatic sulfate reducing bacteria |
|  |  | Desulfarculales |  | Mucus and skin | Aquatic sulfate reducing bacteria. They can eat other bacteria |
|  |  | Desulfuromonadales |  | Mucus and skin | Marine bacteria found in sediments |
|  |  | Desulfobacterales | *Nitrospina* | Water | Nitrifying bacteria |
|  |  | Myxococcales |  | Water | Environmental bacteria |
|  |  | Syntrophobacterales | *Syntrophobacter* | Mucus and skin | Propionate-degrading bacteria [24]. |
|  |  | Thermodesulfobacteriales |  | Mucus and skin | Aquatic bacteria |
|  |  | Campylobacterales | *Arcobacter* | Mucus and skin | *Arcobacter* is an environmental bacteria and is also found as normal microbiota of livestock [25]. |
|  |  |  | *Campylobacter* |  | *Campylobacter* is pathogen in domestic animals and humans. |
|  |  |  | *Sulfurimonas* |  | *Sulfurimonas* has been isolated from polychaetes from hidrotermal vents [26]. |
|  |  |  |  |  |  |
|  |  | Acidithiobacillales | *Acidithiobacillus caldus* | Mucus and skin | Thermophilic environmental bacteria |
|  |  | Alteromonadales | *Alteromonas* | Mucus and skin | *Alteromonas* has been described as pathogen for fish [27]. |
|  |  |  | *Marinobacter* |  | *Marinobacter* and some strains of *Shewanella* have been used to increase fish immune response [28,29]. |
|  |  |  | *Idiomarina* |  | *Idiomarina, Pseudidiomarina homiensis* and *Psychromonas* are marine environmental bacteria. |
|  |  |  | *Pseudidiomarina homiensis* |  |  |
|  |  |  | *Psychromonas* |  |  |
|  |  |  | *Shewanella* |  |  |
|  |  | Caulobacteriales |  | Mucus and skin | Described as normal microbiota of fish gills [30] |
|  |  | Chromatiales |  | Mucus and skin | Environmental Sulfur-oxidizing bacteria |
|  |  | Enterobacteriales | *Baumannia cicadellinicola* | Mucus and skin | *Baumannia cicadellinicola* has been described as a symbiont of insects [31]. |
|  |  |  | *Plesiomonas shigelloides* |  | *Plesiomonas shigelloides* found as normal gut microbiota in fish [32]. |
|  |  |  |  |  |  |
|  |  | Legionellales |  | Mucus and skin | Environmental bacteria and also normal microbiota of animals. |
|  |  | Methylococcales | *Methylobacter whittenburyi* | Mucus and skin | Environmental bacteria |
|  |  | Oceanospirillales | *Marinobacterium* | Mucus and skin | Marine bacteria |
|  |  |  | *Neptunomonas* |  |  |
|  |  |  | *Oleibacter* |  |  |
|  |  | Oceanospirillales | *Halomonas* | Water | Halophilic marine bacteria |
|  |  | Pasteurellales | *Pasteurella* | Water | Some strains of *Pasteurella* and *Haemophilu****s*** have been identified as fish pathogens [33,34]. |
|  |  |  | *Haemophilus* |  |  |
|  |  |  |  |  |  |
|  |  | Pseudomonadales | *Acinetobacter johnsonii* | Water | *Acinetobacter johnsonii* has been described as a fish pathogen [35]. *Moraxella* is an opportunistic pathogen in mammals. |
|  |  |  | *Moraxella* |  |  |
|  |  |  | *Psychrobacter pulmonis* | Mucus and skin | *Psychrobacter pulmonis* was isolated from the lungs of lambs [36]. |
|  |  |  | *Pseudomonas alcaligenes* |  | *Pseudomonas* has been described as normal inhabitant of fish mucus and *Pseudomonas fluorescens* has been isolated from healthy salmon eggs and mucus [37,38]. |
|  |  |  | *Pseudomonas veronii* |  |  |
|  |  |  | *Pseudomonas putrefaciens* |  |  |
|  |  |  | *Pseudomonas fluorescens* |  |  |
|  |  |  |  |  |  |
|  |  | Thiotrichales |  | Mucus and skin | Described from marine sediments |
|  |  | Vibrionales | *Pseudoalteromonas* | Water | Marine environmental bacteria. |
|  |  | Vibrionales | *Vibrio fortis* | Mucus and skin | *Vibrio Fortis* has been found to be pathogenic for seahorses [39]. |
|  |  |  | *Vibrio shilonii* |  | *Vibrio shilonii* has been found to cause coral bleaching [40]. |
|  |  |  | *Vibrio parahaemolyticus,* |  | Some strains of *Vibrio parahaemolyticus* have been suggested as fish pathogens, while others are suggested as protective normal microbiota in fish [41]. |
|  |  |  | *Vibrio alginolyticus* |  | *Vibrio alginolyticus* and *Vibrio fluvialis* appear to be pathogenic for fish [42]. Some Vibrio species are known to be pathogenic for sharks held in captivity, including lemon sharks [43,44] |
|  |  |  | *Vibrio fluvialis* |  |  |
|  |  | Xanthomonadales |  | Mucus and skin | Described as normal microbiota of fish gills [30] |
| Spirochaetas |  | Brachyspirales |  | Mucus and skin | Environmental bacteria |
|  |  | Leptospirales | *Leptospira biflexa* | Water | Some strains of these bacteria may be pathogenic to humans and animals |
|  |  |  |  |  |  |
|  |  |  |  |  |  |
|  |  | Spirochaetales | *Treponema amylovorum* | Water | Found in periodontal lesions [45]. |
|  |  |  |  |  |  |
| Synergistetes |  | Synergistales |  | Water | Marine bacteria |
| Tenericutes |  | Acholeplasmatales | *Acholeplasma* | Water | *Acholeplasma* is found in aquatic environments. *Phytoplasma* is a plant pathogen |
|  |  |  | *Phytoplasma* |  |  |
|  |  | Mycoplasmatales | *Mycoplasma* | Water | Some species are human pathogens |
|  |  | Anaeroplasmatales | *Asteroleplasma* | Mucus and skin | Normal flora of animals and plants |
| Thermi |  | Deinococcales | *Deinococcus* | Mucus and skin | Environmental bacteria |
|  |  | Thermales | *Meiothermus* | Mucus and skin | *Meiothermus* and *Thermus* have been described from hot springs |
|  |  |  | *Thermus* |  |  |
|  |  |  |  |  |  |
| Verrucomicrobia |  | Cerasicoccales |  | Water | Environmental bacteria |
|  |  |  |  |  |  |
|  |  | Opitutales |  | Water | Marine bacteria |
|  |  |  |  |  |  |
|  |  | Pelagicoccales | *Pelagicoccus* | Water | Marine bacteria |
|  |  | Puniceicoccales | *Coraliomargarita* | Water | Marine bacteria |
|  |  | Pedosphaerales |  | Water | Marine bacteria |
|  |  | Chthoniobacterales |  | Water | Environmental bacteria |
|  |  | Verrucomicrobiales | *Akkermansia muciniphila* | Water | *Akkermansia muciniphila* is found in human gut microbiota. |
|  |  |  | *Haloferula* |  | *Haloferula* are halophitic environmental bacteria. *Luteolibacter* is a soil bacterium. |
|  |  |  | *Luteolibacter* |  |  |
|  |  |  |  |  |  |

Protists found in samples of mucus, skin and water from sharks and rays.

| Phylum | Class | Order | Genus and species (if determined) | Type of sample (this study) | Characteristics |
| --- | --- | --- | --- | --- | --- |
|  |  |  |  |  |  |
| Chlorophyta |  | Volvocales | *Carteria cerasiformis* | Mucus and skin | Microalgae |
|  |  |  |  |  |  |
| Cercozoa |  |  |  | Mucus and skin | Protists |
| Euglenophyta |  |  |  | Water | Protists |
| Haptophyta |  | Haptophyceae |  | Mucus and skin | Microalgae |
| Rodophyta |  |  |  | Mucus and skin | Microalgae |
| Stramenopiles |  |  |  | Mucus and skin | Microalgae |
| Streptophyta |  |  |  | Mucus and skin | Microalgae |

Bibliography

1. Ramos CP, Foster G, Collins MD (1997) Phylogenetic Analysis of the Genus *Actinomyces* Based on 16S rRNA Gene Sequences: Description of Arcanobacterium phocae sp. nov., *Arcanobacterium bernardiae* comb. nov., and *Arcanobacterium pyogenes* comb. nov. Int J Syst Bacteriol 47: 46–53. doi:10.1099/00207713-47-1-46.

2. Avendaño-Herrera R, Balboa S, Doce A, Ilardi P, Lovera P, et al. (2011) Pseudo-membranes on internal organs associated with Rhodococcus qingshengii infection in Atlantic salmon (*Salmo salar*). 200-204 p. doi:10.1016/j.vetmic.2010.06.003.

3. Zerihun MA, Hjortaas MJ, Falk K, Colquhoun DJ (2011) Immunohistochemical and Taqman real-time PCR detection of mycobacterial infections in fish. J Fish Dis 34: 235–246. Available: http://www.ncbi.nlm.nih.gov/pubmed/21306590. Accessed 20 October 2016.

4. Kudo T, Hatai K, Seino A (1988) *Nocardia seriolae* sp. nov. Causing Nocardiosis of Cultured Fish. Int J Syst Evol Microbiol 38: 173–178. doi:10.1099/00207713-38-2-173.

5. Kurahashi M, Fukunaga Y, Sakiyama Y, Harayama S, Yokota A (2010) *Euzebya tangerina* gen. nov., sp. nov., a deeply branching marine actinobacterium isolated from the sea cucumber *Holothuria edulis,* and proposal of Euzebyaceae fam. nov., Euzebyales ord. nov. and Nitriliruptoridae subclassis nov. Int J Syst Evol Microbiol 60: 2314–2319. doi:10.1099/ijs.0.016543-0.

6. Albuquerque L, Costa MS da (2014) The Prokaryotes. Rosenberg E, DeLong EF, Lor S, Stackebrand E, Thompson F, editors 357-360 p.

7. Wexler HM (2007) Bacteroides: the good, the bad, and the nitty-gritty. Clin Microbiol 20: 593–621.

8. Sakamoto M, Suzuki N, Matsunaga N, Koshihara K, Seki M, et al. (2009) *Parabacteroides gordonii* sp. nov., isolated from human blood cultures. Int J Syst Evol Microbiol 59: 2843–2847.

9. Moran NA, Tran P, Gerardo NM (2005) Symbiosis and insect diversification: an ancient symbiont of sap-feeding insects from the bacterial phylum Bacteroidetes. Appl Environ Microbiol 71: 8802–8810. Available: http://aem.asm.org/cgi/content/long/71/12/8802. Accessed 20 October 2016.

10. Anand P, Chellaram TC, Kumaran S, Shanthini F (2011) Screening for antibiotic producing marine bacteria against fish pathogens. Int J Pharma Bio Sci 2: B314–B325.

11. Lal S, Tabacchioni S (2009) Ecology and biotechnological potential of *Paenibacillus polymyxa*: a minireview. Indian J Microbiol 49: 2–10. Available: http://www.ncbi.nlm.nih.gov/pmc/articles/PMC3450047/.

12. Olmos J (2014) *Bacillus subtilis* A Potential Probiotic Bacterium to Formulate Functional Feeds for Aquaculture. J Microb Biochem Technol 6: 361–365. Available: http://www.omicsonline.org/microbial-biochemical-technology-abstract.php?abstract_id=31462. Accessed 20 October 2016.

13. Jung A, Rautenschlein S (2014) Comprehensive report of an *Enterococcus cecorum* infection in a broiler flock in Northern Germany. BMC Vet Res 10: 311. Available: http://dx.doi.org/10.1186/s12917-014-0311-7.

14. M. Aguirre, Collins MD (1992) Phylogenetic Analysis of *Alloiococcus otitis* gen. nov., sp. nov., an Organism from Human Middle Ear Fluid. Int J Syst Evol Microbiol 42: 79–83. doi:10.1099/00207713-42-1-79.

15. Zhou M, Yu H, Yin X, Sabour PM, Chen W, et al. (2014) Lactobacillus zeae protects *Caenorhabditis elegans* from enterotoxigenic *Escherichia coli*-caused death by inhibiting enterotoxin gene expression of the pathogen. PLoS One 9.

16. Nho S-W, Shin G-W, Park S-B, Jang H-B, Cha I-S, et al. (2009) Phenotypic characteristics of *Streptococcus iniae* and Streptococcus parauberis isolated from olive flounder (*Paralichthys olivaceus*). FEMS Microbiol Lett 293: 20–27. Available: http://femsle.oxfordjournals.org/content/293/1/20.abstract.

17. Chow S-K, Clarridge JE (2014) Identification and Clinical Significance of *Helcococcus* species, with Description of Helcococcus seattlensis sp. nov. from a Patient with Urosepsis. J Clin Microbiol 52: 854–858. Available: http://www.ncbi.nlm.nih.gov/pmc/articles/PMC3957774/.

18. Lovley DR, Nevin KP (2013) Electrobiocommodities: powering microbial production of fuels and commodity chemicals from carbon dioxide with electricity. Curr Opin Biotechnol 24: 385–390. doi:10.1016/j.copbio.2013.02.012.

19. Flint JF, Drzymalski D, Montgomery WL, Southam G, Angert ER (2005) Nocturnal production of endospores in natural populations of epulopiscium-like surgeonfish symbionts. J Bacteriol 187: 7460–7470. Available: http://jb.asm.org/cgi/content/long/187/21/7460. Accessed 20 October 2016.

20. Hidetoshi Morita, Shiratori C, Murakami M, Takami H, Toh H, et al. (2008) *Sharpea azabuensis* gen. nov., sp. nov., a Gram-positive, strictly anaerobic bacterium isolated from the faeces of thoroughbred horses. Int J Syst Evol Microbiol 58: 2682–2686. doi:10.1099/ijs.0.65543-0.

21. Fukunaga Y, Kurahashi M, Sakiyama Y, Ohuchi M, Yokota A, et al. (2009) *Phycisphaera mikurensis* gen. nov., sp. nov., isolated from a marine alga, and proposal of Phycisphaeraceae fam. nov., hycisphaerales ord. nov. and Phycisphaerae classis nov. in the phylum Planctomycetes. J Gen Appl Microbiol 55: 267–275. doi:10.2323/jgam.55.267.

22. Brucker RM, Harris RN, Schwantes CR, Gallaher TN, Flaherty DC, et al. (2008) Amphibian Chemical Defense: Antifungal Metabolites of the Microsymbiont *Janthinobacterium lividum* on the Salamander P*lethodon cinereus*. J Chem Ecol 34: 1422–1429. Available: http://dx.doi.org/10.1007/s10886-008-9555-7.

23. Thao ML, Moran NA, Abbot P, Brennan EB, Burckhardt DH, et al. (2000) Cospeciation of Psyllids and Their Primary Prokaryotic Endosymbionts. Appl Environ Microbiol 66: 2898–2905. Available: http://aem.asm.org/cgi/content/long/66/7/2898. Accessed 20 October 2016.

24. Boone DR, Bryant MP (1980) Propionate-Degrading Bacterium, S*yntrophobacter wolinii s*p. nov. gen. nov., from Methanogenic Ecosystems. Appl Environ Microbiol 40: 626–632. Available: http://www.ncbi.nlm.nih.gov/pmc/articles/PMC291629/.

25. Lehner A, Tasara T, Stephan R (2005) Relevant aspects of *Arcobacter* spp. as potential foodborne pathogen. Int J Food Microbiol 102: 127–135. doi:10.1016/j.ijfoodmicro.2005.03.003.

26. Takai K, Suzuki M, Nakagawa S, Miyazaki M, Suzuki Y, et al. (2006) S*ulfurimonas paralvinellae* sp. nov., a novel mesophilic, hydrogen- and sulfur-oxidizing chemolithoautotroph within the Epsilon proteobacteria isolated from a deep-sea hydrothermal vent polychaete nest, reclassification of T*hiomicrospira denitrificans* as Su. Int J Syst Evol Microbiol 56: 1725–1733. doi:10.1099/ijs.0.64255-0.

27. McGarey D, Milanesi L, Foley D, Jr Reyes B, Frye L, et al. (1991) The role of motile aeromonads in the fish disease, ulcerative disease syndrome (UDS). Experientia 47: 441–444.

28. Montero J, Gómez-Casado E, García-Alcázar A, Meseguer J, Mulero V (2014) Flagellin from *Marinobacter algicola* and *Vibrio vulnificus* activates the innate immune response of gilthead seabream. Dev Comp Immunol 47: 160–167. doi:10.1016/j.dci.2014.07.003.

29. Tapia-Paniagua ST, Vidal S, Lobo C, Prieto-Álamo MJ, Jurado J, et al. (2014) The treatment with the probiotic *Shewanella putrefaciens* Pdp11 of specimens of S*olea senegalensis* exposed to high stocking densities to enhance their resistance to disease. Fish Shellfish Immunol 41: 209–221. doi:10.1016/j.fsi.2014.08.019.

30. Merrifield D, Rodiles A (2015) The fish microbiome and its interactions with mucosal tissues. Mucosal Health in Aquaculture. pp. 273–289.

31. Cottret L, Milreu PV, Acuña V, Marchetti-Spaccamela A, Stougie L, et al. (2010) Graph-based analysis of the metabolic exchanges between two co-resident intracellular symbionts, *Baumannia cicadellinicola* and *Sulcia muelleri,* with their insect host, *Homalodisca coagulata*. PLoS Comput Biol 6.

32. Sugita H, Shibuya K, Shimooka H, Deguchi Y (1996) Antibacterial abilities of intestinal bacteria in freshwater cultured fish. Aquaculture 145: 195–203. doi:10.1016/S0044-8486(96)01319-1.

33. Bullock GL (1961) A Schematic Outline for the Presumptive Identification of Bacterial Diseases of Fish. Progress Fish-Culturist 23: 147–151. Available: http://dx.doi.org/10.1577/1548-8659(1961)23[147:ASOFTP]2.0.CO.

34. Hawke JP, Plakas SM, Minton RV, McPhearson RM, Snider TG, et al. (1987) Fish pasteurellosis of cultured striped bass (*Morone saxatilis*) in coastal Alabama. Aquaculture 65: 193–204. doi:10.1016/0044-8486(87)90231-6.

35. Kozińska A, Paździor E, Pękala A, Niemczuk W (2014) *Acinetobacter johnsonii* and *Acinetobacter lwoffii* - the emerging fish pathogens. Bull Vet Inst Pulawy 58: 193–199. doi:10.2478/bvip-2014-0029.

36. Vela A, Collins M, Latre M, Mateos A, Moreno M, et al. (2003) *Psychrobacter pulmonis* sp. nov., isolated from the lungs of lambs. Int J Syst Evol Microbiol 53: 415–419. doi:10.1099/ijs.0.02413-0.

37. Cipriano RC, Dove A (2011) Far from superficial: microbial diversity associated with the dermal mucus of fish. Health and diseases of aquatic organisms: bilateral perspectives. MSU Press, East Lansing. 156–167 p.

38. Akinyemi AA, Ekelemu JK, Oyelakin OO, Oloyede AR, Green BM (2016) Molecular characterization of bacteria associated with African catfish *Clarias gariepinus* (Burchell, 1822) from Yewa-Mata station on Yewa River by 16S rRNA gene sequencing method. Glob J Bio-Science Biotechnol 5: 295–300.

39. Wang X, Zhang Y, Qin G, Luo W, Lin Q (2016) A novel pathogenic bacteria (*Vibrio fortis*) causing enteritis in cultured seahorses, *Hippocampus erectus* Perry, 1810. J Fish Dis 39: 765–769. Available: http://www.ncbi.nlm.nih.gov/pubmed/26466548. Accessed 20 October 2016.

40. Kushmaro A, Banin E, Loya Y, Stackebrandt E, Rosenberg E (2001) *Vibrio shiloi* sp. nov., the causative agent of bleaching of the coral *Oculina patagonica*. Int J Syst Evol Microbiol 51: 1383–1388.

41. Austin B, Austin DA (2007) Bacterial Fish Pathogens: diseases of farmed and wild fish. 4th ed. Springer.

42. Zorrilla I, Morinigo MA, Castro D, Balebona MC, Borrego JJ (2003) Intraspecific characterization of *Vibrio alginolyticus* isolates recovered from cultured fish in Spain. J Appl Microbiol 95: 1106–1116. Available: http://doi.wiley.com/10.1046/j.1365-2672.2003.02078.x. Accessed 20 October 2016.

43. Grimes DJ, Colwell RR, Stemmler J, Hada H, Maneval D, et al. (1984) *Vibrio* species as agents of elasmobranch disease. Helgol{ä}nder Meeresuntersuchungen 37: 309–315. Available: http://dx.doi.org/10.1007/BF01989313.

44. Grimes DJ, Stemmler J, Hada H, May EB, Maneval D, et al. (1984) *Vibrio* species associated with mortality of sharks held in captivity. Microb Ecol 10: 271–282. Available: http://www.ncbi.nlm.nih.gov/pubmed/24221148.

45. Wyss C, Choi B, Schüpbach P, Guggenheim B, Göbel U (1997) *Treponema amylovorum* sp. nov., a saccharolytic spirochete of medium size isolated from an advanced human periodontal lesion. Int J Syst Evol Microbiol 47: 842–845. doi:10.1099/00207713-47-3-842.

46. Suau A, Rochet V, Sghir A, Gramet G, Brewaeys S, Sutren M, Rigottier-Gois L, Doré J. 2001. *Fusobacterium prausnitzii* and related species represent a dominant group within the human fecal flora. Syst Appl Microbiol 24: 139-145.
